# The Study of Mechanical Behaviors of *Caprinae* Horn Sheath under Pendulum Impact

**DOI:** 10.3390/polym14163272

**Published:** 2022-08-11

**Authors:** Kang Yang, Nannan Qin, Changgeng Zhou, Bing Wang, Haotian Yu, Haotong Li, Haiyun Yu, Hailiang Deng

**Affiliations:** 1 Key Laboratory of Green Fabrication and Surface Technology of Advanced Metal Materials, Anhui University of Technology, Ministry of Education, Maanshan 243002, China; 2 School of Materials Science and Engineering, Anhui University of Technology, Maanshan 243002, China; 3 State Key Laboratory of Molecular Engineering of Polymers, Fudan University, Shanghai 200433, China; 4 Composite Component Technology Center, Aviation Industry Corporation of China (AVIC) Composite, Corporation Ltd., Beijing 101300, China; 5 South Sichuan Machinery Plant, Luzhou 646000, China

**Keywords:** *Caprinae* horn sheath, mechanical behaviors, multi-scale structure, impact

## Abstract

As a light-weight natural keratin biocomposite, *Bovidae* horn exhibits high mechanical properties and energy absorption. Different to the widely studied horn from subfamily *Bovinae* and *Antilocapridae*, few studies have focused on the horn sheath of subfamily *Caprinae*. In this work, three *Caprinae* horn sheathes from Cashmere goat, White goat and Black sheep were selected. Charpy pendulum impact tests were performed, and the fracture characteristics were evaluated. It was demonstrated that water plays an important role in acquiring balanced dynamic mechanical properties in all *Caprinae* horn sheaths. The hydrated keratin provides large plastic deformation capacity and further gives rise to a gradual generation of micro-cracks. Multi-scale structure including wavy-shaped interface, scattered voids and hierarchical micro-fibre were observed. Such a structure induced complex fracture mechanisms, such as delamination, 90° crack deflection and fibre pull-out, which were probably influenced by interfacial strength. The results are expected to endow the research and thinking of *Bovidae* horn.

## 1. Introduction

Animal horns mainly exist in the *Bovidae* family, including subfamily *Bovinae*, *Caprinae* and *Antilocapridae*. They bear various types of loading in daily fighting and cannot be recovered once damaged. After evolution for millions of years, animal horns have developed the characteristics of light weight, high toughness and impact resistance [1,2,3]. Different kinds of horns exhibit a similar composition: porous bone core and keratin shell, while the keratin shell is the main load-bearing part [4]. Revealing the inherent relationship between the micro-structure and mechanical behavior in keratin shell could provide important inspiration for synthetic materials with lightweight, high-strength and toughness, which has been a research highlight [5,6,7].

The keratin shell of animal horns is derived from the keratinization of dead epithelial cells, mainly including *α*-keratin [8]. The horn sheath can be roughly understood as a model of fiber-reinforced composite formed by embedding keratin fibers with high crystallinity in amorphous keratin matrix [9,10,11]. Keratin is a kind of biomacromolecule [12], which is greatly influenced by water molecule. The role of water is mainly through the following: (1) Weakening the role of molecular chains as a swelling agent; (2) Destroying some hydrogen bonds and increasing the molecule mobility; (3) Forming a loose network structure with keratin matrix as a plasticizer [13,14]. Similar to most biological materials, animal horns show lower stiffness and higher toughness under moisture condition, while higher stiffness and lower toughness under dried condition. Recently, the phenomenon of hydration-assisted shape recovery was observed in the horn sheath [15]. After plastic deformation, the recovery of deformed area could be simulated by water infiltration, then regenerating hydrogen bonds.

Among the horns in subfamily *Antilocapridae*, the research of Bighorn sheep horn has received much attention [16,17,18,19,20]. Its macro-structure could be defined as conical spiral configuration, and the micro-structure features contain tubules, layered lamellae and fiber bundle structure. The multi-scale structural characteristics offer various energy absorption mechanisms, forming multiple crack initiation and increasing crack propagation paths. The regularly arranged hollow tubules with a diameter of 60~200 μm, resulting in a large elastic deformation capacity, effectively dissipating energy and hindering crack propagation [21]. Inspired by the hollow tubules structure with gradient distribution, the impact-resistant motorcycle helmet was manufactured [22]. The outer layer of the helmet absorbs most energy under impact loading, while the inner layer disperses loading and reduces deformation. 3D printing technology is known as an important method to achieve bio-inspired materials with hollow tubules, but it is still difficult to reproduce the superior toughness and impact resistance of animal horns [3,23]. Stress concentration, weak interface strength and residual stress caused by 3D printing processes are important reasons for this challenge. In addition, a recent study reported that whether the hollow tubules structure can play the role of toughening also depends on the tubular distribution, the elastic modulus and yield strength of the intrinsic material [24].

For the horn in subfamily *Bovinae*, such as Yak, Cattle and Buffalo, typical ductile fracture characteristics could be observed in horn sheath previously [5,25,26,27]. The layered structure and wavy-shaped interface have great influence on damage resistance and high toughness. The layered structure and wavy-shaped interface can increase the crack path and energy dissipation [28,29]. The irregular interface between lamella enhances the friction at microscale, leading to a better bonding. Such structure is also conducive to the regulation of moisture, thus forming a balanced mechanical property. It was reported that the critical stress concentration factor of horn sheath could reach ~4.76 under 3-point bending loading [29].

On the other hand, less attention has been paid to the horn of subfamily *Caprinae* until now. A few studies showed that *Caprinae* horn presents outstanding impact resistance, such as Small Tailed Han Sheep (*Ovis aries*) and Deccani breed sheep [30,31]. The microstructure of *Caprinae* horn sheath may be suitable for the design of impact-critical materials, such as vehicle bumpers. Thus, it is worth exploring the dynamic mechanical behaviors and fracture mechanisms of *Caprinae* horn sheath. In this work, three kinds of *Caprinae* including Cashmere goat, White goat and Black sheep were selected. The horn sheath samples were obtained from the directions along the growth at distal location. Both dried and hydrated samples were tested for comparison. Charpy pendulum impact tests were conducted along with the observation with high-speed camera. The fracture morphologies were analyzed by scanning electron microscope. The results are expected to endow the research of horn for bio-inspired design.

## 2. Materials and Methods

### 2.1. Materials

**Figure 1:**
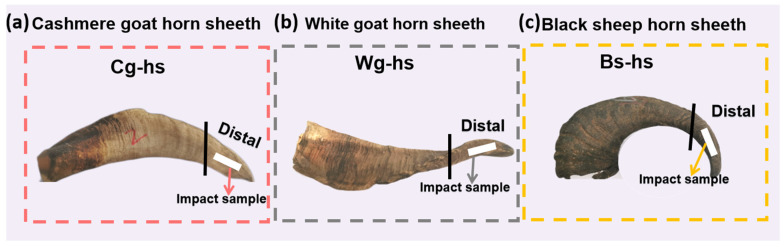
The photographs and impact sampling location of the three *Caprinae* horn sheaths: (**a**) Cashmere goat horn sheath; (**b**) White goat horn sheath; (**c**) Black sheep horn sheath.

The *Caprinae* horns were acquired from Cashmere goat, White goat and Black sheep in this study. All the animals were obtained from China and butchered for dietary reasons. The specimens were cut at the distal location parallel to growth orientation, as shown in Figure 1. Abbreviation of Cg-hs, Wg-hs, Bs-hs were used, representing for the horn sheath of Cashmere goat, White goat and Black sheep. The dried group was acquired by the drying treatment in a chamber at 60 °C for two days. The hydrated group was acquired by immersing in phosphate buffer saline (PBS) solution (0.01 M) for two days. The experiments were conducted within three hours after hydration. Weight uptake after hydration was present in Table 1.

### 2.2. Charpy Pendulum Impact and Flexural Tests

The impact properties of the horn sheaths were measured at room temperature on a Charpy pendulum impact testing machine (HIT50, Zwick Roell, Ulm, Germany) according to International Standard ISO179:1997 Standard. Unnotched specimens (75 × 12.7 × 2 mm) were loaded flat-wise with a 2J hammer at an estimated displacement rate of 3.4 m s^−1^. In situ force-displacement curves were recorded during the impact process. At least five samples were tested for each group. The photographs of the fractured specimens were taken with a digital camera.

The flexural tests of the horn sheaths were conducted under 3-point bending load by an Instron 5565 screw-driven machine. Samples were cut as 30 mm × 8 mm × 3 mm.

### 2.3. High-Speed Camera Observation

A high-speed camera (DIMAX HD, PCO, Kelheim Bavaria, Germany) was adopted to monitor the fracture process during the impact testing. Constellation 120E lamp group is required as light source.

### 2.4. Characterizations of Keratin Structure

The secondary structure was characterized by an ATR-FTIR spectrometer (Thermo Scientific, Waltham, MA, USA) over a wavenumber range of 700–4000 cm^−1^. The peak de-convolution of conformations in the amide I region was conducted using PeakFit (Version 4.12, Systat Software, San José, USA). 

### 2.5. Scanning Electron Microscopy

The microstructures of fracture surfaces were characterized by scanning electron microscopy (SEM, Hitachi S-4800, Tokyo, Japan) with a 3 kV accelerating voltage. The energy-dispersive spectroscopy (EDS) was adopted for element analysis.

## 3. Results and Discussion

### 3.1. Impact Properties of Different Caprinae Horn Sheaths

**Figure 2:**
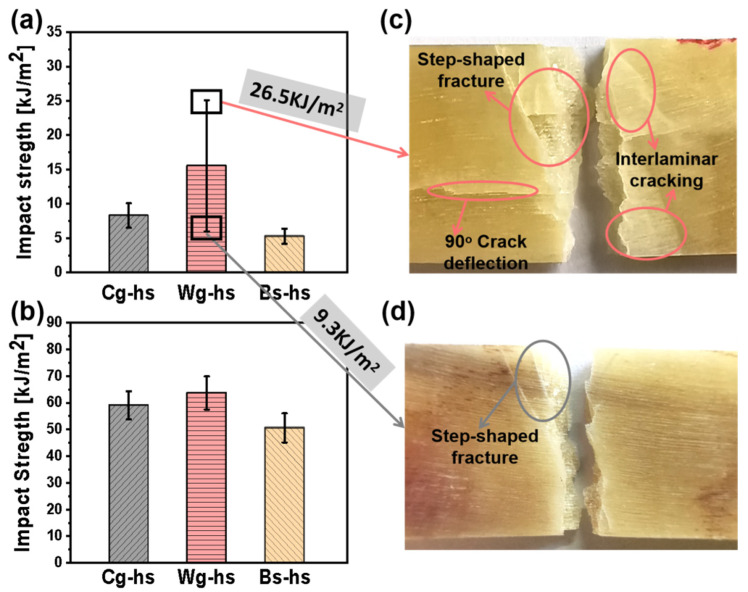
Impact property of three different *Caprinae* horn sheaths. (**a**) Bar chart of impact strength of dried specimens; (**b**) Bar chart of impact strength of hydrated specimens; (**c**) Fracture photograph of the dried Wg-hs sample with high impact strength; (**d**) Fracture photograph of the dried Wg-hs sample with lowest impact strength.

**Figure 3:**
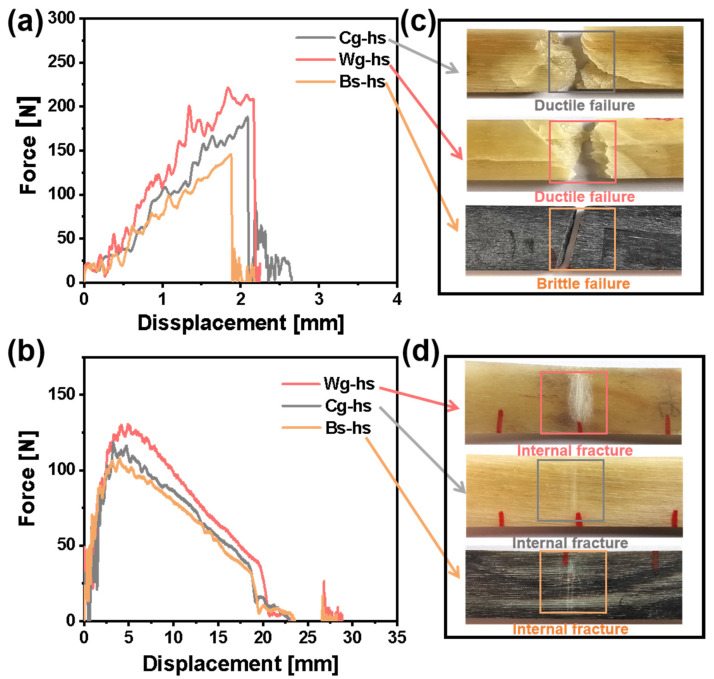
Impact force-displacement curves and fracture photographs of three different *Caprinae* horn sheaths. (**a**) Impact force-displacement curves of dried specimens; (**b**) Impact force-displacement curves of hydrated specimens; (**c**) Fracture photographs of the dried specimens; (**d**) Fracture photographs of the hydrated specimens.

Charpy pendulum impact experiments were conducted on *Caprinae* horn sheath from Cashmere goat, White goat and Black sheep. The results of impact strength and force-displacement curves along the impact process were put in Figure 2 and Figure 3. Apparently, the variable moisture resulted in a great difference of mechanical properties by comparing Figure 2a,b. All the values of impact strength were below 25 kJ m^−2^ in the dried group, but exceed 50 kJ m^−2^ in the hydrated group, indicating the contribution of water to toughness. Similar results could be found from the literatures of other *Bovidae* horn sheath, as the water molecules can act as plasticizers to enhance ductility [3,5]; and the ductility contributes a lot to impact toughness. Similar to *Bovinae* horn sheath, the *Caprinae* could also acquire the balance of strength and toughness through the regulation of water. Compared with the impact strength of engineering plastics and composites, the horn sheath of *Caprinae* with low density (~1.1 g m^−3^) exhibit the advantage of dynamic specific mechanical properties. Additionally, it was interesting that the data dispersity of horn sheath was decreased after immersing it in water. By destroying the original hydrogen bonds and forming a molecular network structure [14], water molecules brought up a well-distributed keratin structure.

**Figure 4:**
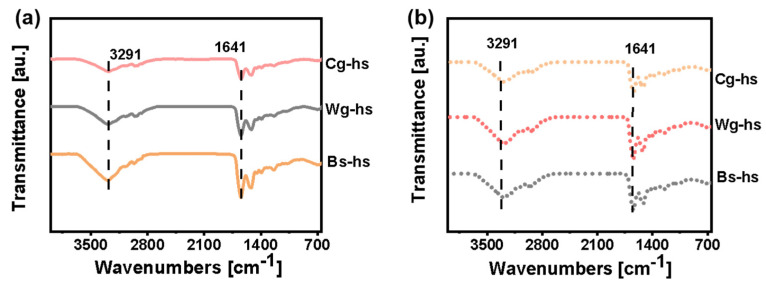
FT-IR curves of different *Caprinae* horn sheaths: (**a**) dried specimens; (**b**) hydrated specimens. Pink line, grey line and orange line indicates for Cg-hs, Wg-hs and Bs-hs, respectively.

Wg-hs exhibited a higher average value of impact strength (~16 kJ m^−2^) than that of Cg-hs and Bs-hs in the dried specimen group. According to the previous works [21,32,33], horn sheath owns multi-scale structure including protein secondary structure, wavy-shaped interface, keratinized fibre and other feature structures (e.g., Tubules). The secondary structure was evaluated by Fourier-transform infrared spectroscopy (Figure 4) and the calculated content of β-sheet were shown in Table 2. It could be seen that the α-helix content is close in different samples, but the β-sheet content of dried Wg-hs is the highest in Table 2 (40.2%), which is related to its high impact strength. In Table 2, the EDS results of Wg-hs showed the highest content of sulfur (5.61%), which is the basis of disulfide bonds to construct β-sheet. The consistent correlation between β-sheet and impact strength could be concluded from the FT-IR and EDS. However, all the other factors also play important roles on mechanical properties. Later, it remains to be studied on the dominate factor which causing the highest performance of Wg-hs among all.

It is worth noting, the data of impact strength in dried Wg-hs was highly dispersed. The photographs of samples with the highest and lowest values (26.5 kJ m^−2^ and 9.3 kJ m^−2^) were summarized in Figure 2c,d. Step-shaped fracture characteristic could be found in both images, but the fracture region was broader and deeper in Figure 2c. The crack propagation induced a large number of interlaminar fracture behaviors, which was based on moderate interfacial strength. Too strong interfacial bonding cannot cause delamination cracking, while too weak interfacial bonding leads to low delamination energy absorption. This could be explained from the theory of fibre composite laminates [34]. In addition, a 90° crack deflection could be observed perpendicular to the loading direction, which was also supposed to offer energy absorption. Such cracks were seldom seen in man-made fiber reinforced composites laminates. A possible strategy was inspired to form such a 90° crack deflection path with consciously construction of inhomogeneous intrafacial bonding for composite laminates.

The force-displacement curves of dried and hydrated samples were presented in Figure 3a,b. All the curves of dried samples showed the characteristics of sudden force decline when the displacement was around 2 mm. Yet, the curves of hydrated samples showed delayed failure after the maximum force point, as the plastic deformation is an important source of toughness. By comparing the two sets of photographs in Figure 3c,d, the cracks throughout the thickness direction could not been found in hydrated samples. Instead, a large number of internal microcracks were observed, which were occurred at the plastic deformation stage undoubtedly. Obviously, the energy absorption of internal microcracks was also based on the large deformation ability of keratin. It was then inferred that the plasticization of water molecule to keratin was more important than the various fracture mechanisms for toughness. In other words, the intrinsic properties of materials are probably crucial to toughness in *Caprinae* horn sheath.

As supplementary information, the flexural properties were also performed for a better understanding of the mechanical behaviors, and the results were shown in Table 3. Obviously, moisture reduces the modulus and flexural strength, which is similar to the trend of the maximum force in the impact performance. Additionally, the dried Wg-hs presented the greatest flexural strength (244.66 MPa), which was higher than that of Cg-hs (181.79 MPa) and Bs-hs (164.69 MPa). The trend of such result is similar to the impact strength.

### 3.2. Fracture Characteristics of Different Caprinae Horn Sheaths

**Figure 5:**
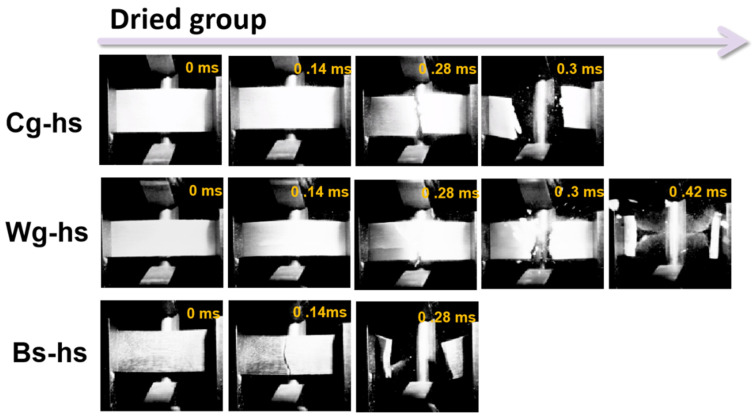
High-speed camera images of impact process for the three *Caprinae* horn sheaths (Dried group).

**Figure 6:**
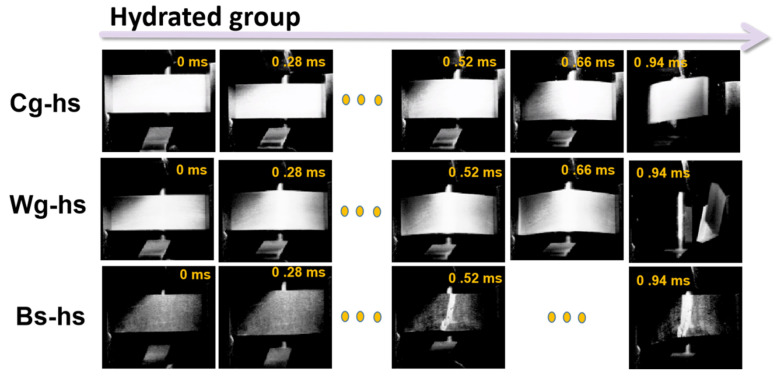
High-speed camera images of impact process for the three *Caprinae* horn sheaths (Hydrated group).

Fracture characteristics were captured on a millisecond scale along the whole impact process by high-speed camera recording, and the results were put in Figure 5 and Figure 6. The fracture process last less than 4.2 ms for dried samples, whereas it increased to 9.4 ms for hydrated samples. The during time for dried Cg-hs, Wg-hs and Bs-hs was 0.3 ms, 0.42 ms and 0.28 ms, positively correlating with the impact strength. Notably, the fracture sequence of dried Wg-hs could be clearly monitored. The 90° crack deflection and delamination cracking occurred before the step-shaped fracture. A few broken fragments splashed out at the end of impact in dried Cg-hs and Wg-hs, whereas it was not reflected in dried Bs-hs with lowest impact strength among all. It was certified that high-speed cameras were an effective method for analyzing fracture behaviors.

**Figure 7:**
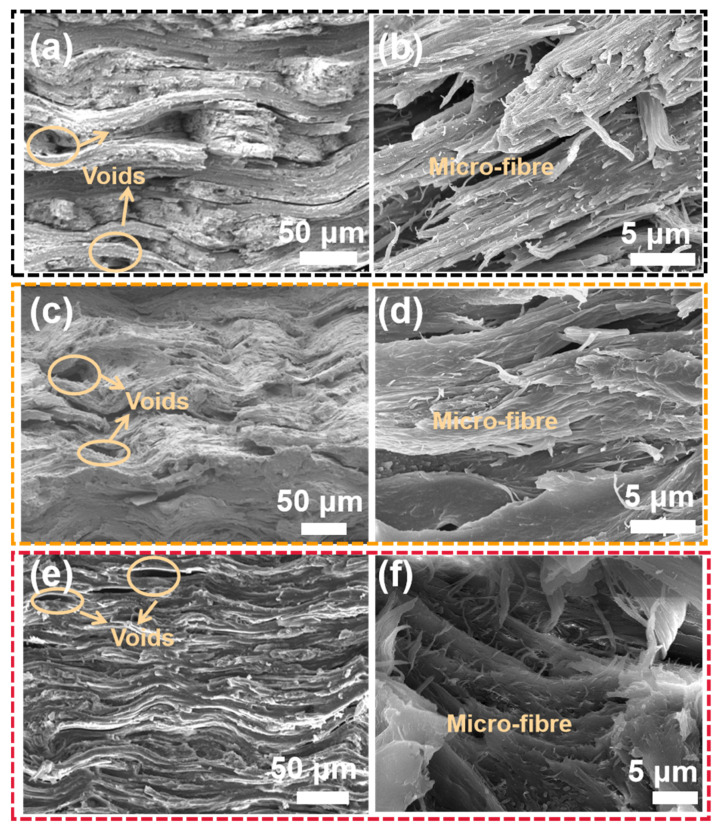
SEM images of brittle fracture with liquid nitrogen of the three *Caprinae* horn sheaths: (**a**,**b**) Cg-hs; (**c**,**d**) Wg-hs; (**e**,**f**) Bs-hs.

In order to understand the micro-structure of the three *Caprinae* horn sheaths, all the samples were immersed into liquid nitrogen and then quickly broken. The low temperature can make the biopolymer more brittle, thus reducing the roughness of the fracture surface, avoiding the interference of the complex crack on the observation of original morphology. Such fracture surfaces were relatively regular and closer to the authentic structure, which are shown in Figure 7. The fiber reinforced composite laminates is applicable to explain the structure of *Caprinae* horn sheath. Wavy-shaped interface, scattered voids and hierarchical micro-fibre were found in all images, proving the similarity of all the three horns qualitatively. These multi-scale structure contributes to strength and toughness undoubtedly. It is known that hollow tubules provide spectacular energy absorption in extensively studied *Antilocapridae* horn sheath and hoof wall [18,35]. Nevertheless, the tubular structure could not be found in *Caprinae*, but wavy-shaped interface and micro-fibre inside deliver the toughening effect. It seems to be closer to *Bovinae* [5], but voids give rise to the particularity of *Caprinae* horn sheath. Synergistic of wavy-shaped interface, scattered voids and hierarchical micro-fibre on toughness is worth pondering carefully in *Caprinae* horn sheath in future.

**Figure 8:**
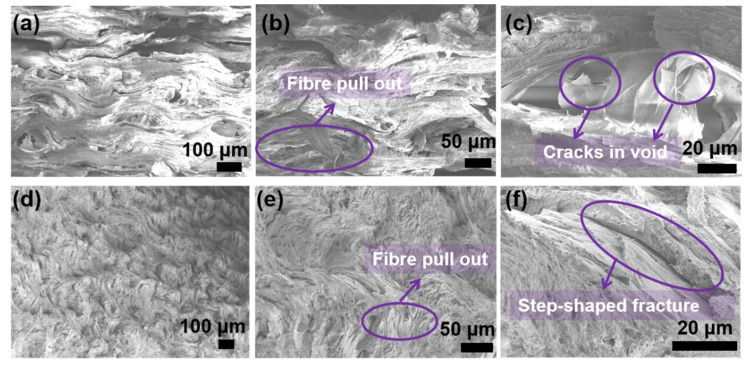
SEM images of impact fracture cross-section of the three dried *Caprinae* horn sheaths: (**a**–**c**) Cg-hs; (**d**–**f**) Wg-hs.

**Figure 9:**
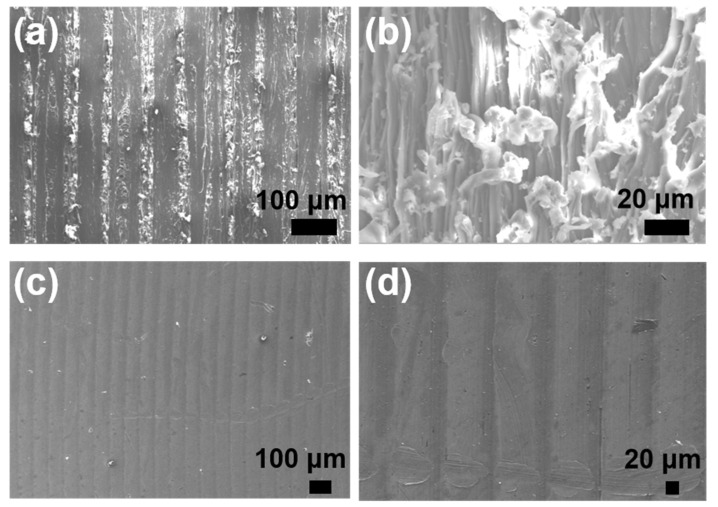
SEM images of surface morphologies after impact tests for the three hydrated *Caprinae* horn sheaths: (**a**,**b**) Cg-hs; (**c**,**d**) Wg-hs.

The typical morphologies of the Cg-hs and Wg-hs were summarized in Figure 8 and Figure 9 after impact tests, represented for the dried and hydrated group, respectively. For the dried group, step-shaped interface lead to the rugged and rough fracture surface, based on a large number of crack deflections from Figure 8a,d. The more microscopic step-shaped fracture was found and displayed in Figure 8f. Fibre fractures and pull-out were discovered in these two specimens from Figure 8b,e. According to the micro-structure analysis above from Figure 7, the fibre failure was not a single crack, but contained a multi-scale fracture which cannot be ignored for toughness. It is worth noting that crack inside the voids could be found from Figure 8c. It is probably a special fracture feature in *Caprinae* different from *Antilocapridae* and *Bovinae*, whereas whether it is an important toughening mechanism remains to be investigated in future. After all, such features were not so abundant from the cross-section surfaces.

For hydrated group in Figure 9, the cross-section surface could hardly emerge without a penetrating fracture. However, some striped micro-cracks were also observed from the surface inspection, which is the extension of internal micro-cracks at the verge. A striped pattern reflected the inside wavy-shaped interface, which generated array fracture characteristics. Definitely, the internal energy absorption behaviors are based on the large deformation ability of hydrated keratin, which could be correlated with the plastic deformation curves in Figure 3b. Clearly, the contribution of water molecules to toughness not only depends on plasticization, but also delivers a gradual generation of micro-cracks in *Caprinae* horn sheath.

## 4. Conclusions

Three specimens of *Caprinae* horn sheaths were chosen to study the impact behavior, and the conclusions are summarized as follows:

(1) All *Caprinae* horn sheaths could acquire a balanced dynamic mechanical property by adjusting water content. The addition of water could also reduce data dispersity of impact property with regulation of a hydrogen bonded network.
(2) Correlation between β-sheet content and impact strength could be found in *Caprinae* horn sheath.
(3) One *Caprinae* (White goat) horn sheath showed distinct fracture characteristics, which was supposed to correlate with the interfacial strength, leading to a various amount of delamination and 90° crack deflection energy absorption.
(4) Wavy-shaped interface, scattered voids and hierarchical micro-fibre constitutes the multi-scale structure of *Caprinae* horn sheath, based on a fiber reinforced composite laminate model.
(5) The large deformation ability of hydrated keratin gives rise to a gradual generation of micro-cracks, contributing to the toughness of *Caprinae* horn sheath.

This study of subfamily *Caprinae* horn sheaths provided some discoveries and approaching thinking about *Bovidae* horn, which was expected to enlighten the design of impact-critical bio-inspired composites.

## Figures and Tables

**Table 1:** Weight uptake of the samples after hydration (unit: g).

Sample	Weight	Sample	Weight	Weight Uptake
Dried Cg-hs	1.31 ± 0.02	Hydrated Cg-hs	1.59 ± 0.03	~0.28
Dried Wg-hs	1.35 ± 0.03	Hydrated Wg-hs	1.62 ± 0.02	~0.27
Dried Bs-hs	1.30 ± 0.03	Hydrated Bg-hs	1.57 ± 0.13	~0.27

**Table 2:** The secondary structure and main element content in different *Caprinae* horn sheaths.

Sample	Secondary Structure		Element			
	α-Helix (%)	β-Sheet (%)	C (%)	N (%)	O (%)	S (%)
Dried Cg-hs	14.9	30.2	55.41 ± 5.4	16.05 ± 4.61	25.21 ± 4.9	3.32 ± 2.33
Dried Wg-hs	13.5	40.2	58.56 ± 7.40	11.47 ± 5.00	24.36 ± 7.62	5.61 ± 4.18
Dried Bs-hs	14.9	30.4	56.60 ± 10.16	12.78 ± 7.83	25.80 ± 4.82	4.83 ± 3.43
Hydrated Cg-hs	14.4	32.4	-	-	-	-
Hydrated Wg-hs	15.0	29.9	-	-	-	-
Hydrated Bs-hs	14.9	30.4	-	-	-	-

Note: The element contents were not obtained as the hydrated samples cannot be observed under scanning electron microscope.

**Table 3:** Flexural properties of different *Caprinae* horn sheaths.

Sample	Elastic Modulus [GPa]	Flexual Strength [MPa]
Dried Cg-hs	3.13 ± 0.09	181.79 ± 23.17
Dried Wg-hs	3.29 ± 0.07	244.66 ± 36.87
Dried Bs-hs	3.03 ± 0.2	164.69 ± 15.22
Hydrated Cg-hs	2.71 ± 0.08	77.74 ± 4.83
Hydrated Wg-hs	2.09 ± 0.03	83.20 ± 21.02
Hydrated Bs-hs	1.47 ± 0.01	82.38 ± 7.15

## Data Availability

Not applicable.
